# Guilt in the Context of Work-Family Conflict, Partner Support, and Life Satisfaction Among Health Professionals

**DOI:** 10.3390/healthcare13172145

**Published:** 2025-08-28

**Authors:** Maria-Manuela Apostol, Magdalena Iorga, Mariana Rotariu

**Affiliations:** 1 Department of Behavioral Sciences, Faculty of Medicine, “Grigore T. Popa” University of Medicine and Pharmacy, 700115 Iasi, Romania; maria_manuela.apostol@umfiaisi.ro; 2 Faculty of Psychology and Education Sciences, “Alexandru Ioan Cuza” University, 700554 Iasi, Romania; 3 Department of Biomedical Sciences, Faculty of Medical Bioengineering, “Grigore T. Popa” University of Medicine and Pharmacy, 700115 Iasi, Romania; mariana.rotariu@umfiasi.ro

**Keywords:** family-work guilt, work-to-family conflict, work-family guilt, medical professionals, life satisfaction, family responsibility, job responsibility

## Abstract

**Background**: Experiencing guilt may adversely affect employee well-being. **Objectives**: This study examined whether guilt proneness and both directions of work-family conflict are associated with domain-specific guilt. **Methods**: Sociodemographic, family-related, and professional data were collected, along with five psychological measures: the Work-Family Conflict Scale, the Five-Item Guilt Proneness Scale, the Work-Family Guilt Scale, the Job Satisfaction Survey, and the Satisfaction with Life Scale. Data were analyzed using SPSS Statistics for Windows, version 29. **Results**: High levels of guilt associated with work-to-family conflict were significantly correlated with lower life satisfaction and higher levels of professional responsibility (time demands, tension, and behaviors) interfering with family life. Family-to-work conflict showed a moderate-to-strong positive correlation with family-work guilt (r = 0.547, *p* < 0.001), and work-to-family conflict showed a moderate-to-strong positive correlation with work-family guilt (r = 0.556, *p* < 0.001). In addition, work-to-family conflict was weakly and negatively associated with life satisfaction (r = −0.234, *p* = 0.012). **Conclusions**: The findings of this cross-sectional study suggest that, among the health professionals surveyed, the degree of work-to-family conflict is influenced by factors such as seniority, work schedule type, and the partner’s involvement in child-rearing.

## 1. Introduction

The relationship between professional and personal life has long been an area of interest for researchers in the organizational psychology field, and this interest has been examined in the wake of increasing professional and familial obligations stemming from dual-working-partner families [1,2,3,4]. With respect to satisfaction with personal and professional life, researchers have identified two types of work-family conflict: work-to-family conflict and family-to-work conflict.

Work-to-family conflict arises when professional demands hinder an individual’s ability to fulfill family responsibilities, whereas family-to-work conflict occurs when family demands impede workplace obligations [5,6,7]. Work-to-family conflict often results in a violation of personal codes [8,9], and it is a type of inter-role conflict wherein the pressures of one’s role in work and family make it impossible for the individual to participate in the activities related to either role [10,11,12]. Specifically, work-to-family conflict occurs when professional role demands prevent individuals from adequately fulfilling their family responsibilities.

Research findings on work-to-family conflict indicated that it is influenced by various factors, including work-related elements such as job type, commitment to working hours, work involvement, role overload, and job flexibility. Additionally, family-related factors, including the number of children, the stage of the life cycle, family involvement, and childcare arrangements, play a role. Individual-related factors, such as life role values, gender role orientation, locus of control, and perfectionism, also contribute to work-to-family conflict [13,14,15].

Numerous studies have demonstrated that elevated levels of work-to-family conflict are negatively correlated with life satisfaction [16,17], job satisfaction [18,19], well-being [20,21,22], the intention to leave an organization, and low levels of marital and family satisfaction. Furthermore, work-to-family conflict is associated with depression, burnout [23,24,25], alcohol abuse, and/or drug use [26,27,28]. Family-to-work conflict occurs when specific family demands hinder an individual’s ability to fulfill work obligations adequately [5,29].

Guilt proneness is a personality trait that reflects an individual’s tendency to experience negative emotions following a mistake or a breach of social or moral norms, even when the individual alone is aware of the transgression. This trait is characterized by the anticipation of tension, remorse, and regret for such violations. Individuals with high guilt proneness scores tend to make fewer unethical decisions in business, engage in fewer delinquent behaviors, and act more honestly in economic decision-making compared to those with lower scores. In professional settings, employees with high guilt proneness are less likely to engage in counterproductive behaviors detrimental to the organization [30,31,32]. The experience of work-to-family guilt arises from the necessity to choose between professional and familial obligations. In circumstances where professional obligations and familial responsibilities are incompatible, individuals are compelled to prioritize one over the other, leading to feelings of guilt due to the discrepancy between personal desires and the choices made [33,34].

Both family-to-work and work-to-family conflicts contribute to feelings of guilt, as they contravene social standards of fulfilling family roles [35,36]. Judge et al. [37] found that individuals experience elevated levels of guilt at work when they perceive significant levels of family-to-work conflict. Family life can interfere with professional duties, hindering effective work performance.

The link between work-to-family conflict and guilt can be elucidated by the Conservation of Resources Theory [38,39], which posits that negative affective outcomes are likely when an individual’s resources are perceived as inadequate to meet demands. Guilt is experienced when an individual recognizes a violation of social–moral norms through behavior deemed inappropriate [40,41]. The medical profession is a very demanding occupation, and due to its specificity (working with patients with physical and emotional disturbance, night shifts, moral dilemmas, sleep deprivation, overloaded schedules, or burnout) healthcare professionals face a high risk of experiencing conflicts. This theory is useful to frame how healthcare professionals experience guilt when there is a strong interference between family demands and job duties.

Work-to-family conflict involves reduced engagement in one role (e.g., family) to meet the demands of another (e.g., professional), behavior that may be perceived as inappropriate and a violation of social norms (e.g., parental involvement in a child’s education). Failures in professional, academic, or athletic domains, as well as breaches of social conventions, are common triggers of guilt [40]. Prior research has established a connection between work-to-family conflict and job satisfaction [42,43,44,45,46].

Lu et al. [47] obtained results that confirmed this association, demonstrating a positive correlation between job demands and work-to-family conflict. Conversely, both job and family demands were positively correlated with the family-to-work conflict dimension. Work-to-family conflict negatively predicted job satisfaction, while family-to-work conflict negatively influenced organizational commitment. An important finding of this study is that organizational policies and practices, such as flexible work schedules, can mitigate work-to-family conflict and the perception of work interfering with family, thereby enhancing job satisfaction and organizational commitment.

Previous research has indicated that one of the consequences of work-family conflict is life satisfaction [17]. Qiu and Fan [48] demonstrated the negative correlation between family flexibility—defined as the capacity for the boundaries of one domain to expand or contract in response to the demands of another domain—and work-to-family conflict, as well as family-to-work conflict. In both instances, these associations were amplified by family permeability, which refers to the degree to which individuals permit elements from one domain (work) to intrude upon another domain (family), such as frequently engaging in work-related phone calls while at home with family. The work-to-family conflict mediated the relationship between family flexibility and life satisfaction. The indirect effect of family flexibility on life satisfaction, mediated by work-to-family conflict, was more pronounced among individuals exhibiting high family permeability.

The objective of this study was to examine the relationship between work-family conflict, specifically its two dimensions: family-to-work conflict and work-to-family conflict, as well as the emotion of guilt associated with these conflicts. We also aimed to determine whether guilt proneness and family-to-work conflict constitute a significant predictive model of guilt related to family-to-work conflict. Similarly, we sought to investigate whether guilt proneness and work-to-family conflict form a significant predictive model of guilt related to work-to-family conflict. Additional analyses were conducted to explore the experience of guilt based on participants’ gender, length of employment, number of hours worked per week, and type of work schedule (regular daytime versus night shifts), as well as the degree of involvement of the partner in the children’s education.

Study Hypotheses:

**H1.**  *There is a significant positive correlation between family-to-work conflict and family-work guilt.*

**H2.**  *There is a significant positive correlation between work-to-family conflict and work-family guilt.*

**H3.**  *Both family-to-work conflict and family-work guilt are significantly and negatively correlated with job satisfaction.*

**H4.**  *Both work-to-family conflict and work-family guilt are significantly and negatively correlated with life satisfaction.*

**H5.**  *Family-to-work conflict and guilt proneness, as well as work-to-family conflict and guilt proneness, establish significant predictive patterns of guilt associated with work-to-family conflict and family-to-work conflict, respectively. In these models, family-to-work conflict and work-to-family conflict exhibit the greatest explanatory power.*

Predictors: family-to-work conflict, work-to-family conflict, guilt proneness.
Criteria: family-work guilt, work-family guilt, job satisfaction, life satisfaction.

## 2. Materials and Methods

### 2.1. Study Design and Participants

A cross-sectional study was carried out using the snowball sampling method. An online questionnaire was created and distributed using Google Forms to doctors and nurses from public and private hospitals in Romania between January and April 2025. The questionnaire could only be completed after the respondent gave consent to participate in the study. No incentives were given to the participants, and they were informed about the data processing, the use of the results, and the fact that they could withdraw from the study at any time. Continuing to complete the questionnaire indicated the participants’ consent to be included in the research. Inclusion criteria: doctors and nurses who were professionally active in public or private hospitals, the state of being married, the state of being a parent, and submitting questionnaires that had been fully filled in. *Exclusion criteria*: questionnaires submitted after the deadline. The questionnaire was distributed for one month, and after the deadline, 114 questionnaires were considered for the research. This cross-sectional design was chosen to capture associations at a single time point. The sample included 114 healthcare professionals (83.3% women, 16.7% men) aged 22–68 years, all married with children, working in public or private hospitals in Romania. The inclusion criteria were theoretically justified by the aim to focus on individuals experiencing both work and family demands; the criteria were verified through self-reports in the online questionnaire. We acknowledge that snowball sampling may introduce selection bias, which is addressed in the limitations section.

### 2.2. Study Instruments

A questionnaire was constructed especially for the present study, and it comprised two parts: In the first one, we collected sociodemographic and family-related data along with information related to their professional life, such as age, sex, marital status, length of experience in the medical field, number of working hours per week, number of children, involvement of the partner in raising children, having a carer for raising children (grandparent or babysitter), and working night shifts.

The second part of the questionnaire included several psychological instruments:

- Work-family conflict was evaluated utilizing the Work-Family Conflict Scale [49], which comprises 18 items, two directional categories (work-family and family-work), and three dimensions for each direction (time, tension, and behavior). The items assess both work-to-family conflict (e.g., the following: the time dimension: “Work keeps me from family activities more than I would like”; the tension dimension: “When I return from work, I am often too exhausted to participate in family activities/responsibilities”; and the behavior dimension: “Behaviors that make me effective at work do not help me be a better parent and spouse”) and family-to-work conflict (e.g., the following: the time dimension: “The time I devote to family responsibilities often interferes with my work responsibilities”; the tension dimension: “Due to stress at home, I am often preoccupied with family problems at work”; and the behavior dimension: “Behaviors that are beneficial to me at home do not seem to be effective at work”). Responses were recorded on a Likert scale ranging from 1 (“strongly disagree”) to 6 (“strongly agree”). To ascertain the extent of work-to-family and family-to-work conflicts, total scores were computed for each dimension (work interference with family and family interference with work). A higher score signifies a greater level of work-family or family-work conflict. The Cronbach’s alpha internal consistency coefficient for the work-family interface factor is 0.84, and for the family-work interface factor, it is 0.85.
- Guilt proneness: Guilt proneness was assessed using the Five-Item Guilt Proneness Scale (GP-5), as developed by Cohen et al. in 2015 [50]. This scale comprises five items, with responses measured on a Likert-type scale ranging from 1 (“very unlikely”) to 5 (“very likely”). An average score is computed across the five items to quantify the respondent’s level of guilt proneness, with higher scores indicating a greater tendency towards guilt. The internal consistency of the scale, as measured using Cronbach’s alpha, was found to be 0.67.
- Work-family guilt and family-work guilt: The Work-Family Guilt Scale (WFGS), developed by McElwain, Korabik, and Chappell (2005) [51], was employed to measure both work-family guilt and family-work guilt. Responses to this scale were recorded on a Likert scale ranging from 1 (“strongly disagree”) to 6 (“strongly agree”). The scale is calculated on a dimensional basis; higher scores reflect greater levels of guilt associated with work-to-family and family-to-work conflict. The internal consistency coefficients, as determined using Cronbach’s alpha, were 0.86 for family-to-work conflict guilt and 0.85 for work-to-family conflict guilt.
- Job satisfaction: Job satisfaction was measured using the Job Satisfaction Survey (JSS), which was developed by Spector (1985) [52]. This instrument consists of 36 items distributed across nine subscales, each containing four items. Responses are rated on a Likert scale ranging from 1 (“strongly disagree”) to 6 (“strongly agree”), with 19 reverse-scored items. The total score, derived from the sum of individual items, serves as an indicator of overall job satisfaction. The internal consistency coefficient, as indicated by Cronbach’s alpha, was 0.91.
- Life satisfaction: Life satisfaction was measured using the Satisfaction with Life Scale (SWLS), developed by Diener et al. (1985) [53]. This scale comprises five items, with responses rated on a Likert-type scale from 1 (“strongly disagree”) to 7 (“strongly agree”). A total score is computed to determine an individual’s level of life satisfaction, with the following interpretations: 31–35 indicates extreme satisfaction; 26–30 represents satisfaction; 21–25 indicates moderate satisfaction; 20 denotes neutrality; 15–19 represents mild dissatisfaction; 10–14 reflects dissatisfaction; and 5–9 indicates extreme dissatisfaction. The Cronbach’s alpha coefficient for internal consistency was 0.87.

### 2.3. Statistical Analysis

The IBM Statistical Package for Social Sciences (SPSS) and Statistics for Windows, version 29—SPSS Inc., Chicago, IL, USA—were used to test the hypotheses [54]. Pearson’s correlation was used to test the first four research hypotheses. Hypothesis 5 was tested using a stepwise multiple regression analysis. The T-test for independent samples was used for comparative statistics. Pearson’s correlation was selected to assess linear associations between variables after confirming normality assumptions. Stepwise multiple regression was applied to identify the strongest predictors, and independent samples T-tests were used for group comparisons. Multicollinearity and distributional assumptions were checked before the analyses.

To assess the normality of the scores distribution, the Kolmogorov–Smirnov (K-S) test was applied with the Lilliefors correction, as the parameters of the distribution (mean and standard deviation) were estimated from the collected data. The Lilliefors correction was applied to adjust the *p*-value, compensating for any potential error introduced by estimating the parameters from the sample data. The results of the K-S test with the Lilliefors correction showed that for the study variables, there was no significant difference from a normal distribution, *p* > 0.05, suggesting that the distribution of the scores can be considered normal. For variables showing deviation from normality, we also examined skewness and kurtosis values. In cases where significant skewness was present, we considered using median and interquartile ranges; however, for consistency and comparability with previous research, we present means and standard deviations while acknowledging this limitation.

The independent variables (predictors) are family-to-work conflict, work-to-family conflict, general predisposition towards guilt, age, and gender.

The dependent variables (criteria) are guilt related to family-work conflict, guilt related to work-family conflict, satisfaction at work, and satisfaction with life.

The dependent variables (criteria) used in the regression analysis are guilt related to work-family conflict (measured using the Work-Family Guilt Scale (WFGS), guilt related to family-work conflict (measured using the Work-Family Guilt Scale (WFGS), job satisfaction (measured using the Job Satisfaction Scale), life satisfaction (measured using the Satisfaction With Life Scale (SWLS).

The independent variables (predictors) included in the regression analysis were work-family conflict (measured using the Multidimensional Work-Family Conflict Scale), family-work conflict (measured using the Multidimensional Work-Family Conflict Scale), general predisposition towards guilt (measured using the Five-Item Guilt Proneness Scale (GP-5)), age, and gender.

The independent variables were chosen based on theoretical relevance and prior literature, which suggests a significant link between work-family conflict, guilt, and job and life satisfaction. Additionally, age and gender were included as demographic factors that might influence these relationships.

The stepwise regression method was used to select significant variables, adding or removing them based on their statistical significance (*p* < 0.05). This process helped simplify the model by keeping only the variables that had a significant impact on the dependent variables. We used the stepwise method to include only relevant predictors, and variables were chosen based on existing theory and observed correlations between variables.

### 2.4. Ethical Approval

The present study was conducted in accordance with the Declaration of Helsinki, and the protocol was approved by the Ethical Committee No. 38/20 January 2025, Prolife Clinics, Iași, Romania. Before starting the survey, the respondents were informed about the purpose of the research, the use of the results, and the confidentiality of the data. Those who agreed to participate could fill in the questionnaires distributed online. No incentives were given to the participants.

## 3. Results

### 3.1. Sociodemographic and Job-Related Data

The sample consisted of 114 medical professionals, all parents, aged 22–68 (41.80 ± 9.50), among whom 95 (83.30%) were women and 19 (16.70%) were men. The larger number of women is specific to the medical profession. All respondents worked in public and private hospitals in Romania and reported a length of experience in the field of about 16.84 ± 9.68 (with a minimum of 1 and a maximum of 41 years). The number of working hours per week was M = 41.48 ± 13.91, and 60 of the participants (52.60%) worked night shifts. More than half of them had a person taking care of their children (grandparent or babysitter), and more than 60% of their partners frequently helped them raise and educate children. Detailed data are presented in Table 1.

### 3.2. Descriptive Statistics—Psychological Instruments

Means and standard deviations were calculated for work-family conflict, family-work conflict, general guilt proneness, guilt related to the work-family conflict, guilt related to the family-work conflict, job and life satisfaction. The results for all five scales used for the present research are presented in Table 2.

### 3.3. Inferential Data Analysis

To test the first four research hypotheses, Pearson correlation analysis was employed. The statistical analysis revealed a positive, moderate-to-strong, and statistically significant correlation between family-to-work conflict and family-work guilt, *r* = 0.547, *p* < 0.001. Similarly, a moderate-to-strong and significant positive correlation was found between work-to-family conflict and work-family guilt, *r* = 0.556, *p* < 0.001.

No significant association was found between family-to-work conflict and job satisfaction, nor between guilt related to family-to-work conflict and job satisfaction (*p* > 0.05). There is a negative, low-intensity, and significant association between work-to-family conflict and life satisfaction, r = −0.234, *p* < 0.012. A negative, low-intensity, and significant correlation was found between guilt related to work-to-family conflict and life satisfaction, r = −0.255, *p* < 0.006. At the same time, a negative correlation of medium intensity that is significant between conflict from work to family and job satisfaction was obtained, r = −0.185, *p* < 0.049.

There is a positive, low-intensity, and significant correlation between the variable general guilt proneness and life satisfaction, r = 0.230, *p* < 0.014, and the variable job satisfaction correlates positively with medium intensity and significantly with the variable life satisfaction (r = 0.352, *p* < 0.001). The detailed correlation results are presented in Table 3.

**Figure 1:**
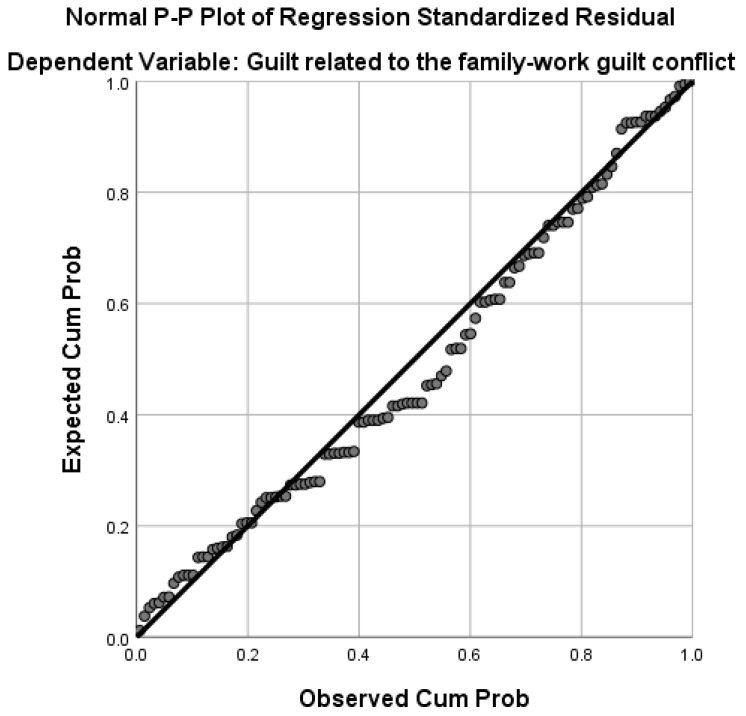
Graphical representation of regression for the variable guilt related to family-to-work conflict.

Hypothesis 5 was tested using stepwise multiple regression analysis. Family-to-work conflict explains 29.9% of the variance of the variable guilt related to family-to-work conflict (F(1,113) = 42.82, *p* < 0.001). High levels of guilt related to conflict from work to family were associated with interference of the dimensions of time, perceived tension, and behavior from the family environment to the professional environment (see Figure 1).

**Figure 2:**
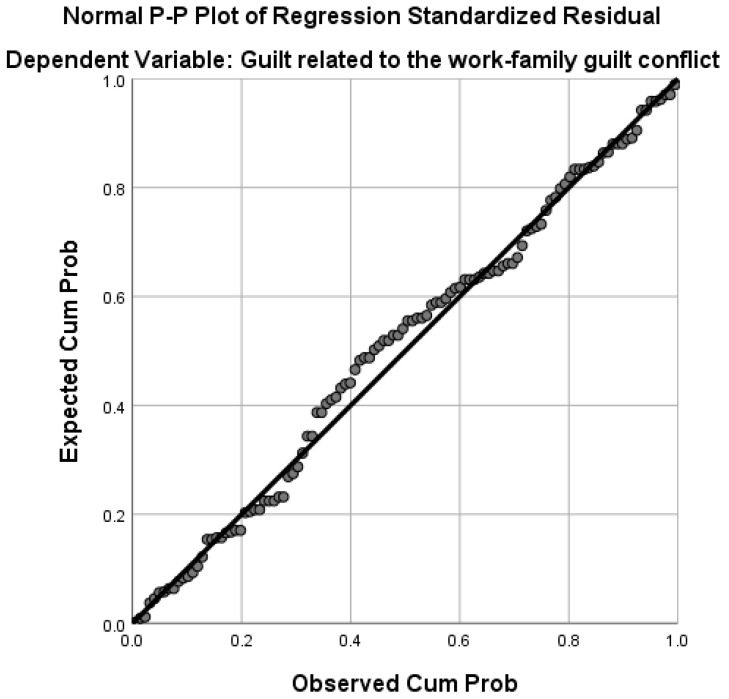
Graphical representation of regression for the variable guilt related to work-to-family conflict.

A percentage of 6.6% of the variance in guilt related to work-to-family conflict is explained by the work-to-family conflict variable (F(1,113) = 7.86, *p* < 0.006). High levels of guilt related to work-to-family conflict are associated with low levels of life satisfaction and high levels of professional responsibilities (time, tension, and behaviors) that interfere with family life (see Figure 2).

An independent samples T-test revealed that participants with shift-based work schedules reported significantly higher levels of work-to-family conflict (M = 33.70, SD = 7.81) than those with regular daytime schedules (M = 30.29, SD = 8.72), t(112) = −2.19, *p* = 0.030. Similarly, with regard to family-to-work conflict, participants employed in the medical field under shift-based schedules (M = 25.55, SD = 8.00) experienced significantly higher levels of conflict compared to their counterparts working regular daytime schedules (M = 21.58, SD = 8.02), t(112) = −2.37, *p* = 0.019.

**Figure 3:**
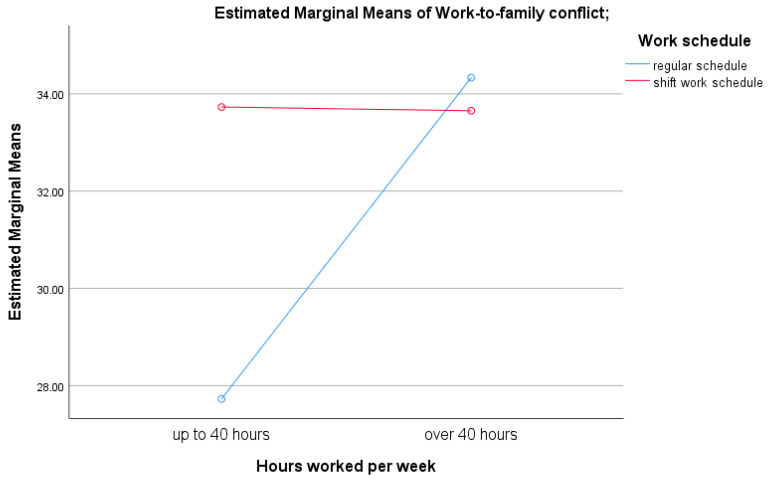
Graphical representation of the interaction effect between the type of work schedule and the number of hours worked per week on work-to-family conflict.

A univariate analysis of variance (ANOVA) further identified a significant interaction effect between the number of hours worked per week and the type of work schedule on levels of work-to-family conflict, F(1,113) = 4.67, *p* = 0.033. Specifically, medical professionals working up to 40 h per week on shift schedules reported significantly greater work-to-family conflict than those with comparable working hours but fixed daytime schedules without night shifts, t(68) = −2.99, *p* = 0.004 (see Figure 3).

**Figure 4:**
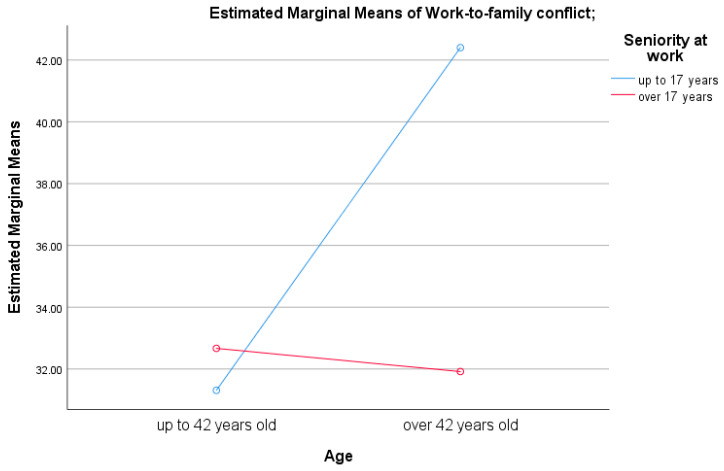
Graphical representation of the interaction effect between seniority and age of subjects on conflict from work to family.

An analysis of variance (ANOVA) revealed a significant interaction effect between years of professional experience and age on levels of work-to-family conflict, F(1,113) = 3.64, *p* = 0.050 (see Figure 4). Specifically, medical personnel with up to 17 years of work experience who were over the age of 42 reported significantly higher levels of work-to-family conflict compared to those with more than 17 years of experience and those aged 42 or younger, t(61) = −2.82, *p* = 0.006.

**Figure 5:**
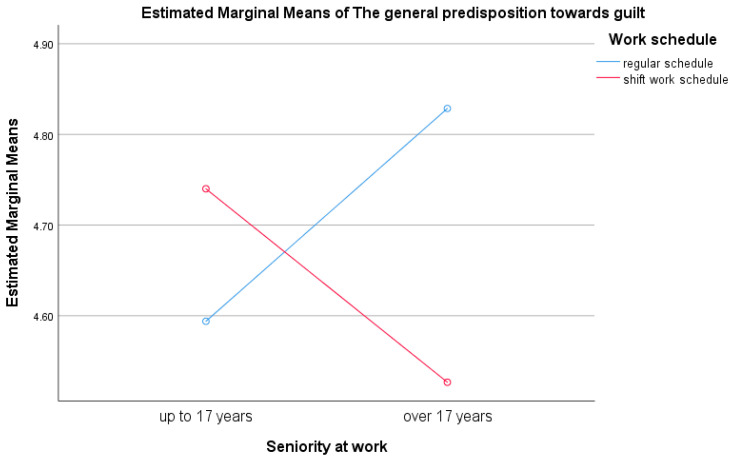
Graphical representation of the interaction effect between seniority and type of work schedule on general guilt proneness.

A significant interaction effect was identified between years of professional experience and the type of work schedule on the dependent variable of general guilt proneness, F(1,113) = 6.20, *p* = 0.014. Specifically, participants with more than 17 years of work experience who followed a regular daytime schedule exhibited significantly higher levels of general guilt proneness compared to their counterparts with equivalent experience who worked night shifts, t(61) = 2.21, *p* = 0.032. These findings are illustrated in Figure 5.

An independent-sample T-test was conducted to examine the impact of a partner’s level of involvement on feelings of guilt related to work-to-family conflict. The results indicated significant differences: greater active participation by a partner in children’s education was associated with higher levels of guilt experienced by the other partner. Specifically, healthcare professionals whose partners were minimally involved in child-rearing reported the lowest guilt regarding work-to-family conflict compared to those whose partners exhibited very high (M = 18.41 ± 6.21, t(34) = −2.35, *p* < 0.024), high (M = 18.08 ± 3.38, t(33) = −3.15, *p* < 0.003), medium (M = 17.60 ± 3.63, t(33) = −2.73, *p* < 0.026), and low (M = 13.50 ± 5.19, t(29) = −2.43, *p* < 0.021) involvement.

Work-to-family conflict is also influenced by the level of a partner’s involvement in child education. Participants who perceived their partners as being very minimally involved in the child-rearing process (M = 34.94, SD = 7.72) reported significantly higher levels of work-to-family conflict compared to those who reported their partners as being highly involved (M = 29.12, SD = 10.21), t(41) = 2.06, *p* = 0.046. Regarding family-to-work conflict, participants whose partners were reported to be highly involved in childcare (M = 20.62, SD = 10.69) experienced significantly lower levels of this form of conflict compared to those whose partners were only slightly involved (M = 28.57, SD = 8.03), t(41) = 2.78, *p* = 0.008.

The level of life satisfaction reported by healthcare professionals appears to be significantly influenced by the degree of their partner’s involvement in child-rearing and education. Greater involvement by the partner is associated with higher levels of life satisfaction. Specifically, medical personnel whose partners are highly involved in raising and educating their children report significantly greater life satisfaction compared to those whose partners are only slightly involved (M = 26.73, SD = 5.34, t(45) = −2.05, *p* = 0.043), moderately involved (M = 25.73, SD = 5.18, t(45) = −2.75, *p* = 0.008), or minimally involved (M = 24.89, SD = 5.10, t(41) = −3.11, *p* = 0.003). These findings suggest that a partner’s active participation in parenting responsibilities has a significant positive impact on the other partner’s subjective well-being and overall perception of life satisfaction.

## 4. Discussion

Statistical analyses supported the first two research hypotheses. Specifically, the results demonstrated a statistically significant, positive, and strong association between family-to-work conflict and feelings of guilt and between work-to-family conflict and feelings of guilt for that associated domain. Based on these results, the demands and expectations of one role (family or work) interfere with the ability to fulfill responsibilities in the other, thereby generating feelings of guilt. Work-family conflicts exist in three distinct forms: time-based conflict, tension-based conflict, and behavior-based conflict. Time-based conflict occurs when the time commitment of one role inhibits participation in the other role. Tension-based conflict arises when the tension experienced in one role has a negative impact on functioning in the other role. Finally, behavior-based conflict occurs when behaviors that are appropriate in one role are incompatible with the behaviors required in the other role [10]. When employees experience higher levels of family-to-work conflict, they are likely to experience higher levels of feelings of guilt and emotional turmoil. Conversely, work-to-family conflict prohibits employees from engaging in family responsibilities, as well as meaningful engagement in activities with family members, e.g., quality time with children or other close family members, which are vital to fulfilling personal and familial goals [1].

Research and theories on work-to-family conflict have identified a number of important consequences for perceptions of work-to-family conflict. Increased work-to-family conflict has been associated with elevated stress levels, increased depression and anxiety rates, anxiety disorders, behavioral disorders, and disorders that increase substance abuse [55,56], dissatisfaction with physical health, hypertension, and the consumption of fatty foods [57,58]. According to the role stress theory—the Michigan Organization Stress Model—individuals have limited energy resources to manage multiple roles, and that balancing these roles tends to involve tension [1,55,56].

Research hypothesis three was not supported because statistical analysis did not reveal any significant negative relationship between guilt related to family-to-work conflict and job satisfaction, nor between family-to-work conflict and job satisfaction. A possible explanation for the lack of an association between family-work conflict/guilt and job satisfaction may lie in the Romanian socio-cultural context. Gender roles and social norms often assign care primarily to women, which can normalize the experience of guilt without directly influencing job satisfaction. In addition, organizational support within health institutions in Romania can cushion the negative impact of family-work conflict on job satisfaction, hiding a direct statistical relationship.

The fourth research hypothesis was supported by the findings. There is a negative association between work-to-family conflict and life satisfaction, as well as between feelings of guilt associated with work-to-family conflict and life satisfaction. Similarly, work-to-family conflict is inversely related to job satisfaction. The consequences of increased work-to-family conflict include decreased satisfaction in personal life and work [59], reduced marital satisfaction, and diminished family satisfaction [60]. In addition to lower job satisfaction, greater work-to-family conflict correlates with higher turnover intentions [61,62]. These results are consistent with prior studies demonstrating the association between work-family conflict and job satisfaction [63]. Job satisfaction reflects the degree to which individuals enjoy their work and represents a subjective assessment, which is closely linked to both productivity and overall well-being.

According to role theory, individuals possess multiple identities, and conflicts can occur when the requirements of these identities interfere with each other [64]. Since family-to-work conflict represents the interference of family with work activities, and work-to-family conflict occurs when work requirements and activities prevent the fulfillment of family responsibilities and affect the quality of family life [65], conflicts should influence satisfaction due to the obstruction of the role fulfillment. In this study, family-to-work conflict was not associated with decreased job satisfaction, but work-to-family conflict was associated with reduced life satisfaction. Another explanation for these results could be related to perfectionism. Without a tendency toward perfectionism, such conflicts may not manifest. Perfectionism generally refers to a tendency to strive to meet high personal standards. Recently, a new perspective on perfectionism has emerged, considering both the positive and negative aspects of perfectionism [66]. This perspective includes three dimensions of perfectionism: high personal standards, a need for order, and the discrepancy between one’s standards and actual performance.

The fifth hypothesis of the study was confirmed, indicating that family-to-work conflict accounts for a significant proportion of the variance in the variable guilt associated with family-to-work conflict. Specifically, a percentage of 6.6% of the variance in guilt related to conflict from work to family is explained by the variable work-to-family conflict.

The second predictive model includes life satisfaction and work-to-family conflict as predictors, which explains 9.9% of the variance in guilt related to work-to-family conflict. Guilt is considered a moral emotion, typically associated with counterfactual thinking—what one should have done [67,68]. As an inward-oriented, self-conscious emotion [40], guilt arises when individuals negatively evaluate their own actions or inactions, contrasting with shame—which, while also a moral emotion, pertains to negative evaluations of oneself as a whole [69]. Furthermore, work-to-family conflict was identified as a negative predictor of job satisfaction.

Guilt associated with work-to-family or family-to-work conflict arises from personal values and an individual’s perspective on their roles, such as being both a medical professional and a parent or partner. Personal values inform one’s objectives and can result in pressures in one area that may hinder the completion of tasks in another. Commitment is also relevant to this discussion, as was previously analyzed in relation to work-family conflict. Commitment describes the investment an individual makes in activities connected to roles within a system [70], including time, physical participation, psychological energy, and cognitive engagement.

The results of our study support those obtained in the studies conducted by Williams and Alliger [71], Barhate et al. [72], and Yasmin & Husna [73], which demonstrated that juggling work and family (the intrusion of one role into the other) and juggling within roles (balancing tasks within a single role) elicited different emotions. They were the first to point out that the demands of work and family must be related to affective state.

The research highlighted a significantly higher level of work-to-family conflict among medical personnel who work in shifts compared to those with a regular, morning work schedule. Shift work involves being on call and spending time away from home, including overnight. Working nights in any profession requires extra effort, even more so in this field. The fatigue accumulated during this shift schedule prevents participants from having the same energy to fulfill their family roles. Regarding family-to-work conflicts, subjects working in the medical field on shifts and on-call duty also experience a significantly higher level of this conflict compared to those in the same field but with a regular daytime schedule. Furthermore, medical staff working up to 40 h a week in shifts report a significantly higher level of work-to-family conflict compared to those working up to 40 h a week on a regular morning schedule. It is understandable that working in shifts, which includes being on call, becomes more tiring and stressful, placing greater pressure on medical personnel.

The results obtained showed that medical personnel with less than 17 years of professional experience and over 42 years of age report a significantly higher level of work-to-family conflict, compared to medical staff with more than 17 years of professional experience and under 42 years of age. This finding is also supported by some previous research [74], while other results have refuted this association [75,76].

The interaction between age and professional experience indicates that older professionals with fewer years of practice report higher levels of conflict. One possible explanation is that people who enter the healthcare sector later may have more difficulty adjusting to demanding schedules, compared to younger peers. Moreover, limited professional experience can reduce the development of effective coping strategies, amplifying the professional-family conflict despite chronological age.

The study identified an interaction effect between years of work experience and type of work schedule on guilt proneness. Subjects with more than 17 years of work experience and a regular morning work schedule showed a significantly higher general guilt proneness compared to those with over 17 years of experience who work in shifts. One possible explanation is that a standard morning work schedule may have prevented these subjects from participating in important family events (such as activities at their children’s schools), which could trigger feelings of guilt. In contrast, those working in shifts have more flexibility, as they can ask colleagues to swap shifts in response to family events. This guilt proneness is seen as a discrepancy between what an individual desires and the choices they have made and is experienced as a negative feeling. It is considered a moral emotion, involving an individual’s awareness that they have violated a social or moral rule through their actions.

Another result of this study that requires analysis concerns the level of involvement of the partner in raising and educating children and its effect on the level of guilt related to work-to-family conflict, the degree of work-to-family conflict, family-to-work conflict, and overall life satisfaction. Regarding the findings in cases of high partner involvement and life satisfaction, these seem to support equity theory, which suggests that people in equitable marriages should be content and satisfied, while those in inequitable marriages should experience stress, with stress increasing as inequity grows [77,78]. Life satisfaction is higher among medical personnel who are in partnerships that are likely to involve cooperation, including constructive behaviors that contribute to the well-being of the relationship, as well as mutual support in family activities, particularly in matters related to the education of children.

Guilt related to family-to-work conflict and guilt related to work-to-family conflict are explained by the predictor of family-to-work conflict. Since work-family conflict involves a perceived failure to meet standards or obligations in the work or family domain—crossing a boundary or violating an inner code—it is clear that guilt results from work-family conflict, with the strength of this emotion depending on the extent to which individuals see themselves as responsible for the conflict. As “a person prone to guilt feels troubled whenever they act contrary to their inner code” [79].

The research data show that, in the case of the medical personnel participating in this study, guilt related to both work-to-family and family-to-work conflict, as well as the level of work-to-family and family-to-work conflict, is influenced by years of work experience, the type of work schedule, and the degree of partner involvement in raising children. The emergence and intensity of these variables can also be explained by the high standards held by these subjects—standards typical of the medical profession, where there is little room for error. While these high standards may seem desirable from the employer’s perspective, many researchers consider perfectionism to be maladaptive [80]. Socially prescribed maladaptive perfectionism has been found to be positively linked to both emotional exhaustion at work and parental stress and negatively linked to both self-satisfaction and life satisfaction [81]. Emotional exhaustion at work and parental stress are representative of, or even consequences of, the perceived work-to-family conflict.

Guilt related to work-to-family conflict is more pronounced among medical personnel whose partners are actively involved in childcare activities. In this context, guilt serves as a negative predictor. One possible explanation is that time devoted to work may prevent an individual from allocating equitable time to family activities. Another explanation could be that emotional exhaustion from work reduces their ability to provide emotional support at home compared to their partner.

The statistical analysis did not identify a significant negative association between job satisfaction and guilt related to family-to-work conflict. However, there is a negative association between life satisfaction and guilt related to work-to-family conflict.

Strengths and limitations: The first limitation of the study is related to the number of participants included in the research, and due to that fact, the result cannot be generalized. The second one is the unbalanced number of men and women included in the study, but this issue is specific to medical professionals, among whom there is a higher number of female doctors and nurses. Therefore, processing comparative results is difficult to take into account, although we did not identify significant differences between female and male individuals regarding work-to-family and family-to-work conflict, findings also observed by other researchers [75,76]. Rich et al. [29] identified that a lack of work-life balance among medical professionals negatively impacted the level of learning and well-being. Women with children were particularly affected. Similar results were highlighted in the studies by Babapour et al. [82] and Ajet et al. [83] that identified higher scores in burnout and lower scores in job satisfaction among female doctors with high family-work conflict. Additional limitations include a potential social desirability bias due to self-report measures, the absence of a longitudinal follow-up to assess causality, and the gender imbalance in the sample, which limits generalizability. The relatively small sample size without an a priori power analysis may also limit statistical power.

Reflections and planning: Achieving a balance between professional and personal life and the standard of “having it all” is difficult to attain. Work is conceived as a social group comprising two or more individuals connected via a common organizational affiliation, as well as individuals bound by a profession, vocation, or other means of livelihood. Similarly, family is also conceived as a social group consisting of two or more people connected by common ancestry, adoption, marriage, or other legally or socially recognized unions. Finding a balance between work and family, as well as proactive participation in family life, is an important prerequisite for life satisfaction.

The study integrates the roles of guilt, work-family conflict, and satisfaction, highlighting the need for organizational interventions such as flexible scheduling and support systems. Future research should employ longitudinal designs or intervention studies with a pre–post evaluation to better understand causal pathways and assess the impact of workplace changes on reducing guilt and improving satisfaction.

From a practical perspective, hospital managers could alleviate guilt and work-family conflict through a combination of organizational and psychological interventions. Structured exchange management, psychological support programs, and family-oriented policies are key directions.

Taking into account the results of this study, several strategies can be proposed: (a) the implementation of hospital shift management policies (predictable rotations, limiting consecutive night shifts); (b) psychological support interventions, such as reflection groups focusing on guilt and preventive burnout programs; (c) and parental support initiatives, including childcare services and policies that promote partner involvement in child-rearing.

## 5. Conclusions

This research investigated the link between work-family conflict—specifically, the two dimensions of family-to-work conflict and work-to-family conflict—and the feeling of guilt experienced in relation to these aspects of work-family conflict. We also highlighted the implications of work-family conflict and the associated guilt for job satisfaction and overall life satisfaction, analyzing whether guilt proneness and family-to-work conflict form a significant predictive model of guilt related to family-to-work conflict. Both work and family play a very important role throughout an individual’s life, and both are necessary to achieve life satisfaction. Our study showed that there is a significant positive association between family-to-work conflict and family-work guilt, as well as a significant positive association between work-to-family conflict and work-family guilt.

## Figures and Tables

**Table 1:** Sociodemographic and job-related data.

Variable	N (%)/M ± ^1^
Sex	
Male	19 (16.70)
Female	95 (83.30)
Age	41.80 ± 9.50
Number of working hours per week	41.48 ± 13.91
Years of experience in the medical field	16.84 ± 9.68
Number of children	
1	66 (57.90)
2	37 (32.50)
3	8 (7)
4	3 (2.60)
The partner helps in raising and educating the children	
Always	24 (21.10)
Frequently	23 (20.20)
Sometimes	23 (20.20)
Rarely	31 (27.20)
Never	13 (11.40)
Having a person to help raise children	59 (51.80)

^1^ Number and percent, mean and standard deviation.

**Table 2:** Results for psychological tools by scale and subscales ^1^.

	Scale	M (SD)
1	Work-family interface	32.08, (8.39)
2	Family-work interface	23.85, (8.17)
3	General guilt proneness	4.65, (0.48)
4	Guilt related to the work-to-family conflict	17.42, (4.78)
5	Guilt related to the family-to-work conflict	7.61, (3.78)
6	Job satisfaction	155.95, (27.15)
7	Life satisfaction	26.48, (5.69)

^1^ Means and standard deviation (M ±).

**Table 3:** Correlation analysis.

	Scale	2	3	4	5	6	7
1	Work-family interface	0.507 **	0.007	0.556 **	0.149	−0.185 *	−0.234 *
2	Family-work interface		−0.012	0.256 **	0.547 **	−0.106	−0.318 **
3	General guilt proneness	−0.012		−0.006	−0.080	0.027	0.230 *
4	Guilt related to the work-to-family conflict	0.256 **	−0.006		0.176	0.877	−0.255 **
5	Guilt related to the family-to-work conflict	0.547 **	−0.080	0.176		−0.995	−0.152
6	Job satisfaction	−0.106	0.027	0.877	−0.095		0.352 **
7	Life satisfaction	−0.318 **	0.230 *	−0.255 **	−0.152	0.352 **	

** Correlation is significant at the 0.01 level (2-tailed); * correlation is significant at the 0.05 level (2-tailed).

## Data Availability

Data are available upon request from the corresponding author.

## References

[1] Hammer L.B., Kossek E.E., Anger W.K., Bodner T., Zimmerman K.L. (2011). Clarifying work-family intervention processes: The roles of work-family conflict and family-supportive supervisor behaviors. J. Appl. Psychol..

[2] Annink A., Den Dulk L., Steijn B. (2016). Work-family conflict among employees and the self-employed across Europe. Soc. Indic. Res..

[3] Ilies R., Schwind K.M., Wagner D.T., Johnson M.D., DeRue D.S., Ilgen D.R. (2007). When can employees have a family life? The effects of daily workload and affect on work-family conflict and social behaviors at home. J. Appl. Psychol..

[4] Moreira A., Encarnação T., Viseu J., Au-Yong-Oliveira M. (2023). Conflict (work-family and family-work) and task performance: The role of well-being in this relationship. Adm. Sci..

[5] Borelli J.L., Nelson-Coffey S.K., River L.M., Birken S.A., Moss-Racusin C. (2017). Bringing work home: Gender and parenting correlates of work-family guilt among parents of toddlers. J. Child Fam. Stud..

[6] Borelli J.L., Nelson S.K., River L.M., Birken S.A., Moss-Racusin C. (2017). Gender differences in work-family guilt in parents of young children. Sex Roles.

[7] Aybas M., Özçelik G., Uyargil C. (2022). Can decent work explain employee-level outcomes? The roles of work-family and family-work conflict. Sustainability.

[8] Judge T.A., Ilies R., Scott B.A. (2006). Work-family conflict and emotions: Effects at work and at home. Pers. Psychol..

[9] Marôco A.L., Nogueira F., Gonçalves S.P., Marques I.C. (2022). Work-family interface in the context of social responsibility: A systematic literature review. Sustainability.

[10] Greenhaus J.H., Beutell N.J. (1985). Sources and conflict between work and family roles. Acad. Manag. Rev..

[11] La Torre G., Grima D., Romano F., Polimeni A. (2021). Perceived work ability and work-family conflict in healthcare workers: An observational study in a teaching hospital in Italy. J. Occup. Health.

[12] Pien L.C., Cheng W.J., Chou K.R., Lin L.C. (2021). Effect of work-family conflict, psychological job demand, and job control on the health status of nurses. Int. J. Environ. Res. Public Health.

[13] Ahmad A. (2008). Job, family and individual factors as predictors of work-family conflict. J. Hum. Resour. Adult Learn..

[14] Perreault M., Power N. (2023). Work-life balance as a personal responsibility: The impact on strategies for coping with interrole conflict. J. Occup. Sci..

[15] de Sousa C., Viseu J., Pimenta A.C., Vinagre H., Ferreira J., Matavelli R., José H., Sousa L., Romana F.A., Valentim O. (2024). The effect of coping on the relationship between work-family conflict and stress, anxiety, and depression. Behav. Sci..

[16] Michel J.S., Rotch M., Shifrin N., DeBaylo P., Lennox A. (2025). Work-family conflict and enrichment predict work and family negative and positive affect and (sometimes) vice versa: A prospective analysis. Int. J. Stress Manag..

[17] Sousa C., Pinto E., Santos J., Gonçalves G. (2020). Effects of work-family and family-work conflict and guilt on job and life satisfaction. Pol. Psychol. Bull..

[18] Sousa C., Gato C., Gonçalves G., Sousa A. (2022). Work-family conflict and guilt: Effects on well-being and career satisfaction. Occupational and Environmental Safety and Health IV.

[19] Chen Z., Promislo M.D., Powell G.N., Allen T.D. (2024). Examining the aftermath of work-family conflict episodes: Internal attributions, self-conscious emotions, family engagement, and well-being. Psychol. Rep..

[20] Boyar S.L., Mosley D.C. (2007). The relationship between core self-evaluations and work and family satisfaction: The mediating role of work-family conflict and facilitation. J. Vocat. Behav..

[21] Qu H., Zhao X.R. (2012). Employees’ work-family conflict moderating life and job satisfaction. J. Bus. Res..

[22] Yang H., Zhao X.R., Ma E. (2024). A dual-path model of work-family conflict and hospitality employees’ job and life satisfaction. J. Hosp. Tour. Manag..

[23] Mumu J.R., Tahmid T., Azad M.A.K. (2020). Job satisfaction and intention to quit: A bibliometric review of work-family conflict and research agenda. Appl. Nurs. Res..

[24] Yildiz B., Yildiz H., Ayaz Arda O. (2021). Relationship between work-family conflict and turnover intention in nurses: A meta-analytic review. J. Adv. Nurs..

[25] Frank E., Zhao Z., Fang Y., Rotenstein L.S., Sen S., Guille C. (2021). Experiences of work-family conflict and mental health symptoms by gender among physician parents during the COVID-19 pandemic. JAMA Netw. Open.

[26] Kuntsche S., Kuntsche E. (2021). Drinking to cope mediates the link between work-family conflict and alcohol use among mothers but not fathers of preschool children. Addict. Behav..

[27] Roberto K.J., Taylor J.F. (2022). Alcohol and cigarette use affecting the relationship between work-life conflict and physical health. Community Work Fam..

[28] Miller B.K., Wan M., Carlson D., Kacmar K.M., Thompson M. (2022). Antecedents and outcomes of work-family conflict: A mega-meta path analysis. PLoS ONE.

[29] Rich A., Viney R., Needleman S., Griffin A., Woolf K. (2016). ‘You can’t be a person and a doctor’: The work-life balance of doctors in training—A qualitative study. BMJ Open.

[30] Cohen T.R., Panter A.T., Turan N. (2012). Guilt proneness and moral character. Curr. Dir. Psychol. Sci..

[31] Wahlers D.E., Hart W., Lambert J.T. (2024). Judging the guilt of the un-guilty: The roles of “false positive” guilt and empathy in moral character perception. J. Exp. Soc. Psychol..

[32] Barr P. (2022). Dimensions of the Burnout Measure: Relationships with shame-and guilt-proneness in neonatal intensive care unit nurses. Aust. Crit. Care.

[33] Lent R.W., Brown S.D., Wang R.J., Cygrymus E.R., Moturu B.P. (2024). Looking ahead, looking around, and looking to others: Identifying core proactive behaviors in the quest for career sustainability. J. Career Assess..

[34] Claxton G., Hosie P., Sharma P. (2022). Toward an effective occupational health and safety culture: A multiple stakeholder perspective. J. Saf. Res..

[35] Livingston B.A., Pichler S., Kossek E.E., Thompson R.J., Bodner T. (2022). An Alpha, Beta and Gamma approach to evaluating occupational health organizational interventions: Learning from the measurement of work-family conflict change. Occup. Health Sci..

[36] O’Neill M. (2024). Guilt work and family. Int. J. Employ. Stud..

[37] Livingston B.A., Judge T.A. (2008). Emotional responses to work-family conflict: An examination of gender role orientation among working men and women. J. Appl. Psychol..

[38] Hobfoll S.E. (2021). Conservation of resources theory: Its implication for stress, health, and resilience. The Oxford Handbook of Stress, Health, and Coping.

[39] Hobfoll S.E. (1989). Conservation of resources: A new attempt at conceptualizing stress. Am. Psychol..

[40] Tangney J.P., Burggraf S.A., Wagner P.E. (1995). Shame-proneness, guilt-proneness, and psychological symptoms. Self-Conscious Emotions: The Psychology of Shame, Guilt, Embarrassment, and Pride.

[41] Aarntzen L., Derks B., Van Steenbergen E., Van Der Lippe T. (2023). When work-family guilt becomes a women’s issue: Internalized gender stereotypes predict high guilt in working mothers but low guilt in working fathers. Br. J. Soc. Psychol..

[42] Eby L.T., Casper W.J., Lockwood A., Bordeaux C., Brinley A. (2005). Work and family research in IO/OB: Content analysis and review of the literature (1980–2002). J. Vocat. Behav..

[43] Ergeneli A., Ilsev A., Karapınar P. (2009). Work-family Conflict and Job Satisfaction Relationship: The Roles of Gender and Interpretive Habits. Gend. Work Organ..

[44] Pressley C., Garside J. (2023). Safeguarding the retention of nurses: A systematic review on determinants of nurse’s intentions to stay. Nurs. Open.

[45] Marufu T.C., Collins A., Vargas L., Gillespie L., Almghairbi D. (2021). Factors influencing retention among hospital nurses: Systematic review. Br. J. Nurs..

[46] Maniscalco L., Enea M., De Vries N., Mazzucco W., Boone A., Lavreysen O., Baranski K., Miceli S., Savatteri A., Fruscione S. (2024). Intention to leave, depersonalisation and job satisfaction in physicians and nurses: A cross-sectional study in Europe. Sci. Rep..

[47] Lu L., Kao S.-F., Chang T.-T., Wu H.-P., Cooper C.L. (2011). Work/family demands, work flexibility, work/family conflict, and their consequences at work: A national probability sample in Taiwan. Int. Perspect. Psychol. Res. Pract. Consult..

[48] Qiu L., Fan J. (2015). Family boundary characteristics, work-family conflict and life satisfaction: A moderated mediation model. Int. J. Psychol..

[49] Carlson D.S., Kacmar K.M., Williams L.J. (2000). Construction and initial validation of a multidimensional measure of work-family conflict. J. Vocat. Behav..

[50] Cohen T.R., Kim Y., Panter A.T. (2014). The five-item guilt proneness scale (GP-5). Differences.

[51] McElwain A., Korabik K., Chappell D.B. The work-family guilt scale. Proceedings of the Annual Meeting of the Canadian Psychological Association.

[52] Spector P.E. (1994). Job Satisfaction Survey.

[53] Diener E.D., Emmons R.A., Larsen R.J., Griffin S. (1985). The satisfaction with life scale. J. Personal. Assess..

[54] Hayes A.F. (2009). Beyond Baron and Kenny: Statistical Mediation Analysis in the New Millennium. Commun. Monogr..

[55] Jones M.C., Smith K., Johnston D.W. (2005). Exploring the Michigan model: The relationship of personality, managerial support and organizational structure with health outcomes in entrants to the healthcare environment. Work Stress.

[56] Adams A., Golsch K. (2023). Consequences of work-to-family conflicts for parental self-efficacy—The impact of gender and cultural background in Germany. J. Fam. Issues.

[57] Wu H.P., Wang Y.M. (2022). Women’s work-family conflict and its consequences in commuter marriages: The moderating role of spouses’ family commitment in a dyad analysis. Front. Psychol..

[58] Allen T.D., Armstrong J. (2006). Further examination of the link between work-family conflict and physical health: The role of health-related behaviors. Am. Behav. Sci..

[59] Kossek E.E., Lee K.H., Aldag R.J. (2017). Work-family conflict and work-life conflict. Oxford Research Encyclopedia of Business and Management.

[60] Minnotte K.L., Minnotte M.C., Bonstrom J. (2015). Work-family conflicts and marital satisfaction among US workers: Does stress amplification matter?. J. Fam. Econ. Issues.

[61] De Vries N., Boone A., Godderis L., Bouman J., Szemik S., Matranga D., De Winter P. (2023). The race to retain healthcare workers: A systematic review on factors that impact retention of nurses and physicians in hospitals. J. Health Care Organ. Provis. Financ..

[62] De Vries N., Lavreysen O., Boone A., Bouman J., Szemik S., Baranski K., Godderis L., De Winter P. (2023). Retaining healthcare workers: A systematic review of strategies for sustaining power in the workplace. Healthcare.

[63] Taşdelen-Karçkay A., Bakalım O. (2017). The mediating effect of work-life balance on the relationship between work-family conflict and life satisfaction. Aust. J. Career Dev..

[64] Baldwin J.H., Ellis G.D., Baldwin B.M. (1999). Marital satisfaction: An examination of its relationship to spouse support and congruence of commitment among runners. Leis. Sci..

[65] Maglalang D.D., Sorensen G., Hopcia K., Hashimoto D.M., Katigbak C., Pandey S., Takeuchi D., Sabbath E.L. (2021). Job and family demands and burnout among healthcare workers: The moderating role of workplace flexibility. SSM-Popul. Health.

[66] Slaney R.B., Rice K.G., Mobley M., Trippi J., Ashby J.S. (2001). The revised almost perfect scale. Meas. Eval. Couns. Dev..

[67] Ogunfowora B., Nguyen V.Q., Lee C.S., Babalola M.T., Ren S. (2023). Do moral disengagers experience guilt following workplace misconduct? Consequences for emotional exhaustion and task performance. J. Organ. Behav..

[68] Mazzone A., Yanagida T., Camodeca M., Strohmeier D. (2021). Information processing of social exclusion: Links with bullying, moral disengagement and guilt. J. Appl. Dev. Psychol..

[69] Harris N. (2003). Reassessing the dimensionality of the moral emotions. Br. J. Psychol..

[70] Rothbard N.P. (2001). Enriching or depleting? The dynamics of engagement in work and family roles. Adm. Sci. Q..

[71] Williams K.J., Alliger G.M. (1994). Role stressors, mood spillover, and perceptions of work-family conflict in employed parents. Acad. Manag. J..

[72] Barhate B., Hirudayaraj M., Dirani K., Barhate R., Abadi M. (2021). Career disruptions of married women in India: An exploratory investigation. Hum. Resour. Dev. Int..

[73] Yasmin T., Husna C.A. (2020). Familial support as a determinant of women career development: A qualitative study. Asian J. Soc. Sci. Leg. Stud..

[74] Burke R.J., Greenglass E.R. (1999). Work-family conflict, spouse support, and nursing staff well-being during organizational restructuring. J. Occup. Health Psychol..

[75] Kinnunen U., Geurts S., Mauno S. (2004). Work-to-family conflict and its relationship with satisfaction and well-being: A one-year longitudinal study on gender differences. Work Stress.

[76] Kinnunen U., Mauno S. (1998). Antecedents and outcomes of work-family conflict among employed women and men in Finland. Hum. Relat..

[77] Zheng G., Lyu X., Pan L., Chen A. (2022). The role conflict-burnout-depression link among Chinese female health care and social service providers: The moderating effect of marriage and motherhood. BMC Public Health.

[78] Ali P.A., McGarry J., Maqsood A. (2022). Spousal role expectations and marital conflict: Perspectives of men and women. J. Interpers. Violence.

[79] Demos J. (1996). Shame and guilt in early New England. The Emotions.

[80] Robakowska M., Tyrańska-Fobke A., Walkiewicz M., Tartas M., Ślęzak D., Tomczak W., Balwicki Ł., Zorena K., Jałtuszewska S. (2021). PerfectionisM and Burnout in health care Professionals. Emerg. Med. Serv..

[81] Wang Q., Wu H. (2021). The mediating role of self-compassion and its components in the relationship between maladaptive perfectionism and life satisfaction among Chinese medical students. Curr. Psychol..

[82] Babapour A.R., Gahassab-Mozaffari N., Fathnezhad-Kazemi A. (2022). Nurses’ job stress and its impact on quality of life and caring behaviors: A cross-sectional study. BMC Nurs..

[83] Ajet G.S., Offong R.E., Ajayi M.P., Iruonagbe T.C., Amoo E.O. (2019). Work-family conflict and burnout among female medical doctors in selected hospitals Abuja. IOP Conference Series: Materials Science and Engineering.

